# Cool and warm ionotropic receptors control multiple thermotaxes in *Drosophila* larvae

**DOI:** 10.3389/fnmol.2022.1023492

**Published:** 2022-11-14

**Authors:** Alisa A. Omelchenko, Hua Bai, Emma C. Spina, Jordan J. Tyrrell, Jackson T. Wilbourne, Lina Ni

**Affiliations:** School of Neuroscience, Virginia Tech, Blacksburg, VA, United States

**Keywords:** temperature sensation, *Drosophila* larvae, ionotropic receptor (IR), temperature preference, thermotaxis

## Abstract

Animals are continuously confronted with different rates of temperature variation. The mechanism underlying how temperature-sensing systems detect and respond to fast and slow temperature changes is not fully understood in fly larvae. Here, we applied two-choice behavioral assays to mimic fast temperature variations and a gradient assay to model slow temperature changes. Previous research indicates that Rhodopsin 1 (Rh1) and its phospholipase C (PLC) cascade regulate fast and slow temperature responses. We focused on the ionotropic receptors (IRs) expressed in dorsal organ ganglions (DOG), in which dorsal organ cool-activated cells (DOCCs) and warm-activated cells (DOWCs) rely on IR-formed cool and warm receptors to respond to temperature changes. In two-choice assays, both cool and warm IRs are sufficient for selecting 18°C between 18°C and 25°C but neither function in cool preferences between 25°C and 32°C. The Rh1 pathway, on the other hand, contributes to choosing preferred temperatures in both assays. In a gradient assay, cool and warm IR receptors exert opposite effects to guide animals to ∼25°C. Cool IRs drive animals to avoid cool temperatures, whereas warm IRs guide them to leave warm regions. The Rh1 cascade and warm IRs may function in the same pathway to drive warm avoidance in gradient assays. Moreover, IR92a is not expressed in temperature-responsive neurons but regulates the activation of DOWCs and the deactivation of DOCCs. Together with previous studies, we conclude that multiple thermosensory systems, in various collaborative ways, help larvae to make their optimal choices in response to different rates of temperature change.

## Introduction

Humans and all other organisms are constantly confronted with and respond to internal and ambient temperature variations throughout their lives. Molecular thermoreceptors detect such changes and help animals maintain their body temperature or avoid potentially harmful thermal extremes (Morrison and Nakamura, 2019; Xiao and Xu, 2021). Fruit flies possess multiple thermosensory systems that are activated by different temperature ranges, including noxious cold, innocuous cool, innocuous warm, and noxious hot (Barbagallo and Garrity, 2015; Li and Gong, 2017; Xiao and Xu, 2021). The activation of thermosensory systems also depends on the rate of temperature change. Adult flies, for example, have two warm sensing pathways, both of which are activated at ∼25°C. The anterior cells in the brain rely on a transient receptor potential ion channel, TRPA1, to guide flies to slowly leave warm temperatures when they are exposed to a shallow gradient (Hamada et al., 2008). Each arista contains three heating and three cooling cells (Gallio et al., 2011). The warm receptor in heating cells is a gustatory receptor paralogue, GR28B (D), whereas the cool receptor in cooling cells is made up of three ionotropic receptors (IRs), IR25a, IR93a, and IR21a. Both GR28B(D) and IRs are necessary to direct flies to avoid a sudden increased temperature (Ni et al., 2013; Budelli et al., 2019). However, only IRs, but not GR28B(D), drive cool avoidance in response to a fast temperature decrease (Budelli et al., 2019).

In early third-instar fly larvae, Rhodopsin 1 (Rh1) and its phospholipase C (PLC) cascade, including Gq α subunit, PLC, and TRPA1, detect both fast and slow temperature changes (Kwon et al., 2008; Shen et al., 2011; Sokabe et al., 2016). On a temperature gradient, the *Rh1* mutant larvae accumulated in warm regions, suggesting this pathway is necessary for selecting the optimal temperature in response to slow temperature changes (Sokabe et al., 2016). On the other hand, mutations eliminating any component in this cascade impaired cool preferences in the two-choice assay between 18°C and 24°C, indicating their roles in responding to sudden temperature variations (Kwon et al., 2008; Shen et al., 2011). Rh5/6 and their signaling cascade function in a different larval stage (the late third instar) and guide animals to 18°C on a shallow gradient (Sokabe et al., 2016).

Fly larvae possess three cool-activated cells (DOCCs) and two warm-activated cells (DOWCs) in each dorsal organ ganglion (DOG) (Klein et al., 2015; Hernandez-Nunez et al., 2021). Both DOCCs and DOWCs depend on IRs to detect temperature changes. They share the same co-receptor IRs, IR25a and IR93a, but express distinct tuning receptors. IR21a is the tuning receptor for cool receptors, and IR68a is for warm receptors (Knecht et al., 2016; Ni et al., 2016; Hernandez-Nunez et al., 2021). Optogenetics shows that both DOCCs and DOWCs are sufficient to drive avoidance of stimuli (Klein et al., 2015; Hernandez-Nunez et al., 2021; Tyrrell et al., 2021). Although DOCCs and cool IR receptors are necessary for cool avoidance when animals travel along a shallow temperature gradient (Klein et al., 2015; Tyrrell et al., 2021), their role in responding to a sudden temperature change is unclear. Furthermore, the behavioral consequences of DOWCs and warm IRs to different temperature stimuli have not been studied.

Here, we discovered the behavioral importance of cool and warm IR receptors using different thermotactic behavioral assays. Two-choice assays were used to mimic fast temperature variations and a gradient assay to model slow temperature changes. When a sudden temperature increase occurs, cool and warm IRs are both sufficient for selecting 18°C between 18°C and 25°C, but neither function in cool preferences between 25°C and 32°C. The Rh1 pathway, on the other hand, contributes to choosing preferred temperatures in both temperature ranges. On a shallow temperature gradient, cool and warm IRs have the opposite effects. While cool IRs guide animals to avoid cool temperatures, warm IRs direct them to leave warm regions. Mutations affecting both warm IRs and the Rh1 cascade resulted in a more severe defect accumulating in warm regions, suggesting they function in the same pathway. Furthermore, IR92a also contributes to the selection of the cool temperature specifically in the two-choice assay between 18°C and 25°C. Although neither DOCCs nor DOWCs express IR92a, it may regulate the activation of DOWCs and the deactivation of DOCCs.

## Materials and methods

### Fly strains

*Canton-S* (*CS*) was used as the *wild type* (*wt*) control. The following flies were previously described: *ninaE*^17^ (O’Tousa et al., 1985), *Gq*^1^ (Scott et al., 1995) (RRID:BDSC_42257), *norpA^P24^* (Ostroy and Pak, 1974) (RRID:BDSC_9048), *trpA1*^ins^** (Rosenzweig et al., 2008), *Ir21a^Δ1^* (Ni et al., 2016), *Ir25a*^2^ (Benton et al., 2009) (RRID:BDSC_41737), *Ir68a^MB^* (Knecht et al., 2017) (RRID:BDSC_26031), *Ir76b*^1^ (Zhang et al., 2013) (RRID:BDSC_51309), *Ir76b*^2^ (Zhang et al., 2013) (RRID:BDSC_51310), *Ir92a^MI^* (RRID:BDSC_43017), *Ir93a^MI^* (Knecht et al., 2016) (RRID:BDSC_42090), *Ir25a-Gal4* (Silbering et al., 2011) (RRID:BDSC_41728), *Ir93a-Gal4* (Sanchez-Alcaniz et al., 2018), *Ir92a-Gal4* (Silbering et al., 2011) (RRID:BDSC_41733), *UAS-GFP* (*p{10XUAS-IVS-Syn21-GFP-p10}attP2*) (Pfeiffer et al., 2012), *{Ir25a^+^}* (*BAC{Ir25a^+^}*) (Chen et al., 2015), *UAS-Ir93a* (Knecht et al., 2016), *UAS-TNT* (*UAS-TeTxLC*) (Sweeney et al., 1995), *Ir68a-Gal4 (Knecht et al., 2017)*, *Ir21a*^123^ (Ni et al., 2016), and *UAS-GCaMP6m* (*p{20XUAS-IVS-GCaMP6m}attP40*) (Chen et al., 2013) (RRID:BDSC_42748).

*Ir21a-Gal80* was created by subcloning the *Ir21a* promoter region into *pBPGAL80Uw-6* (Addgene plasmid #26236) (Pfeiffer et al., 2010; Ni et al., 2016). *UAS-Ir92a* contains the *Ir92a* primary transcript including introns (chromosome 3R: 20,338,973-20,433,902), which was cloned into *pUAST-attB* (Bischof et al., 2007). Both constructs were integrated into attP2 (Markstein et al., 2008) (RRID:BDSC_8622).

### Larval thermotactic assays

Flies were maintained at 25°C under 12-h light/dark cycles and early third-instar [72 h after egg laying (AEL)] larvae were collected. The gradient assay was described in detail previously (Tyrrell et al., 2021). Briefly, a temperature gradient of 13 ± 1–31 ± 1°C was created. Every 1°C on the temperature gradient was located and demarcated, resulting in 18 temperature zones. Larvae were given 10–15 min to distribute along the temperature gradient. The larval number in each temperature zone was then counted, and the fraction of the larval number in each temperature zone to the total number was calculated.

Two-choice assays were performed at 20°C as described with some modifications (Kwon et al., 2008; Tyrrell et al., 2021). The apparatus was created by two aluminum plates (30.5 × 30.5 × 0.6 cm). To create 25 ± 1 or 32 ± 1°C, an aluminum plate was placed on a hot plate (SP88850200, Fisher Scientific). The hotplate was set to about 32°C and 45°C to create gel surface temperatures of 25 ± 1 and 32 ± 1°C, respectively. To create the gel surface temperature of 18 ± 1°C, two fly vial reload trays (59–207, Genesee Scientific) were used. One tray was placed upside down besides the hot plate used to create 25 ± 1°C. The other was placed on top of it and filled with ice to create an ice tray. The ice tray was half full and the ice surface to the tray top edge was about 2 cm. An aluminum plate was placed on the top of the ice tray. The side adjacent to the other aluminum plate was aligned with the side of the ice tray. The two aluminum plates were separated by ∼0.16 cm to form a release zone. If two aluminum plates were not exactly level, a few small pieces of paper were used to raise the ice tray or the hot plate. To set up the two-choice assay between 25°C and 32°C, two hot plates were placed side by side and the release zone was created in the middle of two hot plates. To make the agar gel, 800 mL of 3% agar gel was autoclaved and poured into a Pyrex tray (39 × 26 × 6 cm). A stainless steel metal ruler was used to resize the agar gel to ∼25.4 × 24 × 0.9 cm after it had solidified for about 30 min. The agar gel was flipped upside down and placed on the surface created by the two aluminum plates to allow the release zone in the middle of the gel. The temperature was monitored before each trial using a surface temperature probe (50-993-321, Fisher Scientific) and thermometer (15-078-187, Fisher Scientific). All experiments were conducted between 9:00 a.m. and 6:00 p.m. at dim ambient light (<10 lux). A *wt* control was run at the beginning of daily experiments. Water was gently sprayed between trials to moisten the agar surface. 15–30 larvae were placed at the release zone and given 2 min to wander. The number of larvae was counted on each side, and the cool preference index (PI) was calculated using the following formula:


$$\mathrm{PI}=\frac{\begin{array}{c} \left(\mathrm{number}\,\mathrm{of}\,\mathrm{larvae}\,\mathrm{on}\,\mathrm{the}\,\mathrm{cool}\,\mathrm{side}\right)\\ -\left(\mathrm{number}\,\mathrm{of}\,\mathrm{larvae}\,\mathrm{on}\,\mathrm{the}\,\mathrm{warm}\,\mathrm{side}\right) \end{array}}{\begin{array}{c} \mathrm{total}\,\mathrm{number}\,\mathrm{of}\,\mathrm{larvae} \end{array}}$$

### Calcium imaging

Calcium imaging of larval neurons to temperature changes was performed as described (Tyrrell et al., 2021). Briefly, larvae were immobilized in 1 × PBS between a glass slide and a glass coverslip (22 × 40 mm). Imaging was performed on a Zeiss LSM 880 with a z-axis piezo stage (432339-9000-000, Wienecke & Sinske). Airyscan Fast mode and Definite Focus were applied to correct focus drift. Z-stacks were acquired at 512 × 512 resolution and a 1.5 zoom using a 25 × water objective. A thermoelectric cooler was built by attaching a Peltier (30 × 30 mm, TE-127-1.0-0.8, TE Technology) to a heat sink (12.9 × 5.5 cm, modified from ATS2193-ND, Digi-Key) and powered using a 2A power supply (CSI1802X, Circuit Specialists). The Peltier was placed directly on the slide covering the larvae to deliver temperature stimuli. The temperature range was about 12°C–28°C. The temperature was monitored using a type-N thermocouple microprobe (IT-24P, Physitemp) that was mounted near the larvae, and recorded by a data acquisition device (USB-TEMP, Measurement Computing) and DAQami software (Measurement Computing). The temperature was maintained at room temperature for 30 s. Then, three cycles of temperature fluctuations were applied by decreasing the temperature to 12°C–16°C for 30 s and increasing it back to 26°C–28°C for 30 s. Images were analyzed and ΔF/F was calculated using ImageJ plugins TACI (Omelchenko et al., 2022) and TrackMate (Tinevez et al., 2017).

### Immunostaining

Immunostaining was performed as described (Kang et al., 2011). The following antibodies were used: guinea pig anti-IR21a (1:100) (Budelli et al., 2019), rabbit anti-IR93a (1:100) (Knecht et al., 2016), guinea pig anti-IR25a (1:100) (Benton et al., 2009), mouse anti-GFP (1:500; Roche), goat anti-guinea pig Cyanine Cy™3 (1:100; Jackson ImmunoResearch), goat anti-rabbit Alexa Fluor^®^ 647 (1:100; Jackson ImmunoResearch), and goat anti-mouse CF™ 488A (1:200; Sigma-Aldrich). IR21a, IR93a, and IR25a antibodies were kind gifts from Dr. Richard Benton.

### Statistical analysis

Statistical details of experiments were described in figure legends. The normality of distributions was assessed by the Shapiro-Wilk W test (*P* ≤ 0.05 rejected normal distribution). Statistical comparisons of normally distributed data were performed by the two-tailed unpaired *t*-test or, for multiple comparisons, the Ordinary one-way ANOVA test followed by the Tukey test. For data that did not conform to a normal distribution, statistical comparisons were performed by the Mann-Whitney test. Data analysis was performed using GraphPad Prism 9.

## Results

### The Rh1 pathway and ionotropic receptors expressed in dorsal organ ganglions play different roles in two-choice thermotaxes

**FIGURE 1:**
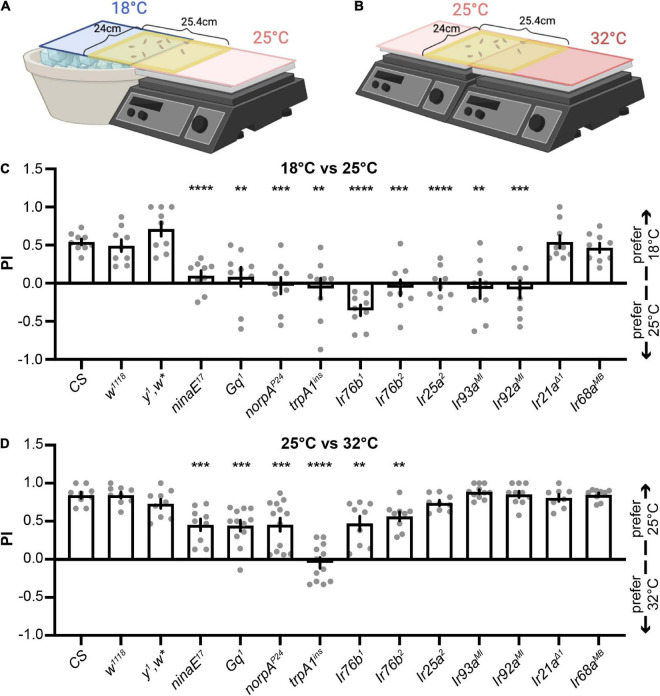
The Rh1 pathway and IRs expressed in DOGs regulate different thermotactic behaviors. **(A,B)** Schematics of two-choice assays (created with BioRender). Early third-instar larvae were released in the middle of a testing gel and allowed to choose between 18°C and 25°C **(A)** or between 25°C and 32°C **(B)** for 2 min. The number of larvae in each temperature zone was counted and used to calculate the preference index (PI) to cool temperatures. **(C,D)** PIs of indicated genotypes obtained from larval thermotactic assays between 18°C and 25°C **(C)** or between 25°C and 32°C **(D)**. *n* = 8–13; data represent means ± s.e.m; **P < 0.01, ***P < 0.001, and ****P < 0.0001, comparing PI with the corresponding *CS*, the Welch’s test, except the Mann–Whitney test for the comparison of *CS* and *Ir21a^Δ1^* in **(C)**, *CS* and *norpA^P24^* in **(D)**, and *CS* and *Ir68a^MB^* in **(D)**.

We set up two apparatuses to perform two-choice assays for investigating larval temperature preferences (Figures 1A,B). Early-third instar larvae were released from the middle and given 2 min to select between 18°C and 25°C (Figure 1A) or between 25°C and 32°C (Figure 1B). Preference indexes (PIs) to cool temperatures were calculated. *Wild type* (*wt*) larvae, including *Canton-S* (*CS*), *w*^1118^, and y^1^,w*, preferred 18°C between 18°C and 25°C (Figure 1C, Supplementary Figure 1, and Supplementary Video 1). Rh1 (encoded by *ninaE*) and its PLC signaling pathway, including the Gq α subunit, PLC (encoded by *norpA*), and TRPA1, were necessary for cool preferences between 18°C and 25°C (Figure 1C). In the two-choice assay between 25°C and 32°C, *wt* larvae (*CS*, *w*^1,118^, and y^1^,w*) preferred 25°C (Figure 1D and Supplementary Video 2). The choice of 25°C was impaired in mutants eliminating the Rh1 pathway, indicating it was also required to select the cool temperature between 25°C and 32°C (Figure 1D). Please note the unique phenotype of the *trpA1*^ins^** mutant. While *ninaE^17^, Gq^1^*, and *norpA^P24^* reduced cool preferences between 25°C and 32°C, they did not eliminate it. *trpA1*^ins^**, however, abolished this temperature preference (Figure 1D). These findings imply that TRPA1 may function beyond the Rh1 signaling pathway to control temperature preferences.

Larval DOGs contain temperature-sensitive neurons that express IRs to detect different temperatures (Knecht et al., 2016; Ni et al., 2016; Hernandez-Nunez et al., 2021). Thus, we examined the role of IRs expressed in DOGs in two-choice assays (Sanchez-Alcaniz et al., 2018). IR25a and IR93a are co-receptor IRs for both cool and warm receptors (Knecht et al., 2016; Ni et al., 2016; Hernandez-Nunez et al., 2021). *Ir25a*^2^ and *Ir93a^MI^* mutants lost their preferences to the cool temperature between 18 and 25°C (Figure 1C), but neither had defects in the assay between 25 and 32°C (Figure 1D). The *Ir92a^MI^* mutant exhibited similar phenomena to *Ir25a*^2^ and *Ir93a^MI^*, suggesting IR25a, IR93a, and IR92a are specifically necessary to select the cool temperature between 18 and 25°C, but not between 25°C and 32°C (Figure 1D). IR21a is the tuning IR for cool receptors, and IR68a for warm receptors (Ni et al., 2016; Hernandez-Nunez et al., 2021). Neither *Ir21a^Δ1^* nor *Ir68a^MB^* showed defects in selecting 18°C between 18°C and 25°C (Figure 1C) or 25°C between 25°C and 32°C (Figure 1D). IR76b is another co-receptor IR for chemosensation (Ni, 2020). Two mutant alleles of *Ir76b* were tested. Both showed defects in choosing cool temperatures in two-choice assays between 18°C and 25°C (Figure 1C) as well as between 25°C and 32°C (Figure 1D). Among all *Ir* mutants tested in this study, *Ir76b* mutants displayed unique phenotypes that impaired temperature choices in both assays. IR76b is a broadly expressed co-receptor IR (Ni, 2020). Since no tuning IRs expressed in DOGs exhibited similar phenotypes to *Ir76b* mutants, IR76b may play an indirect role in regulating thermotaxes or function outside of DOGs. IR25a, IR93a, and IR92a, on the other hand, exhibited the same phenotype. Therefore, we focused on IR25a, IR93a, and IR92a and investigated their unique function in controlling temperature preferences between 18°C and 25°C in following studies.

### IR93a and IR92a are not expressed in the same neurons

**FIGURE 2:**
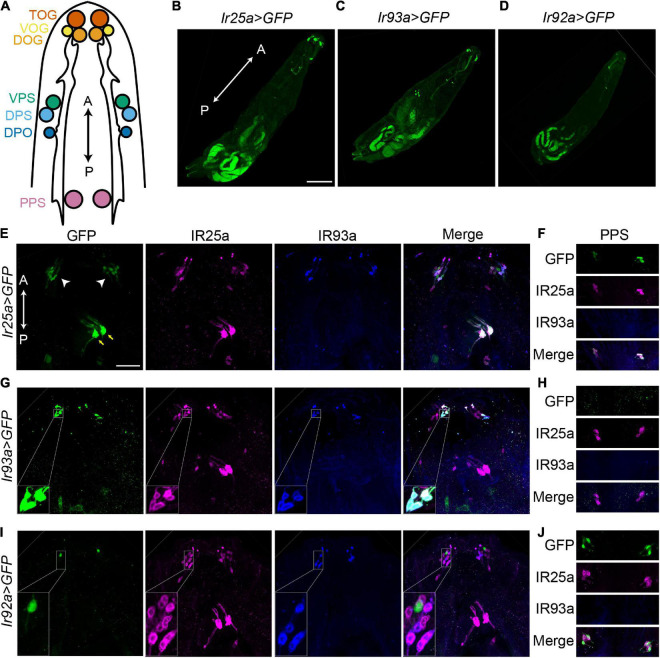
IR93a and IR92a are not expressed in the same neurons. **(A)** The top view of a larval anterior part to show chemosensory organs, including the terminal organ ganglion (TOG), dorsal organ ganglion (DOG), ventral organ ganglion (VOG), dorsal pharyngeal sensilla (DPS), ventral pharyngeal sensilla (VPS), dorsal pharyngeal organ (DPO), and posterior pharyngeal sensilla (PPS). The orientation is shown by double headed arrows. A, anterior; P, posterior. **(B–D)** The expression of *Ir25a-Gal4* **(B)**, *Ir93a-Gal4* **(C)**, and *Ir92a-Gal4* **(D)** in first instar larvae. Scale bars, 200 μm. **(E–J)** Immunostaining of GFP [green; *Ir25a > GFP* **(E,F)**, *Ir93a > GFP* **(G,H),** and *Ir92a > GFP* **(I,J)**], IR25a (magenta), and IR93a (blue) in the anterior part of third instar larvae. **(E,G,I)** include TOG, DOG, VOG, DPS, VPS, and DPO. White arrowheads: TOG, DOG, and VOG; yellow arrows: DPS, VPS, and DPO. **(F,H,J)** show PPS. Scale bars, 50 μm. **(G)** Inset: Co-localization of *Ir93a-Gal4*, an IR93a antibody, and an IR25a antibody in DOGs. **(I)** Inset: *Ir92a-Gal4* is not co-expressed with an IR93a antibody or an IR25a antibody in DOGs. *Ir25a > GFP*: *Ir25a-Gal4; UAS-GFP*; *Ir93a > GFP*: *Ir93a-Gal4/UAS-GFP*; and *Ir92a > GFP*: *Ir92a-Gal4/UAS-GFP*.

Given that *Ir25a^2^, Ir93a*^MI^**, and *Ir92a^MI^* mutants displayed similar behavioral phenomena, we examined whether IR25a, IR93a, and IR92a were expressed in the same cells. *Ir25a-Gal4* and an IR25a antibody showed that IR25a was broadly expressed in anterior chemosensory organs, including the terminal organ ganglion (TOG), DOG, ventral organ ganglion (VOG), dorsal pharyngeal sensilla (DPS), ventral pharyngeal sensilla (VPS), dorsal pharyngeal organ (DPO), and posterior pharyngeal sensilla (PPS) (Figures 2A,B,E,F). In each DOG, *Ir93a-Gal4* and an IR93a antibody labeled five neurons that also expressed IR25a (Figures 2C,G,H). Although *Ir93a-Gal4* was detected in several cells in the middle of the body, these cells were labeled by neither *Ir25a-Gal4* nor *Ir92a-Gal4* and thus were not further investigated (Figures 2B–D).

IR92a antibodies were not available. We attempted to generate IR92a antibodies against the peptide ELEFIDKYMDKKKQEVLMD using two rabbits, but failed. Thus, we completely relied on *Ir92a-Gal4* to understand the expression of IR92a. *Ir92a-Gal4* was expressed in one neuron in each DOG, which was not labeled by IR25a or IR93a antibodies (Figure 2I). The IR25a antibody, however, labeled *Ir92a-Gal4* positive neurons in PPS (Figure 2J). Since we were unable to detect co-expression of IR93a and IR92a, we hypothesized that IR93a and IR92a functioned in different cells to control temperature preferences. As a result, we investigated their roles independently.

### Both cool and warm IR receptors are sufficient for cool preferences between 18°C and 25°C

**FIGURE 3:**
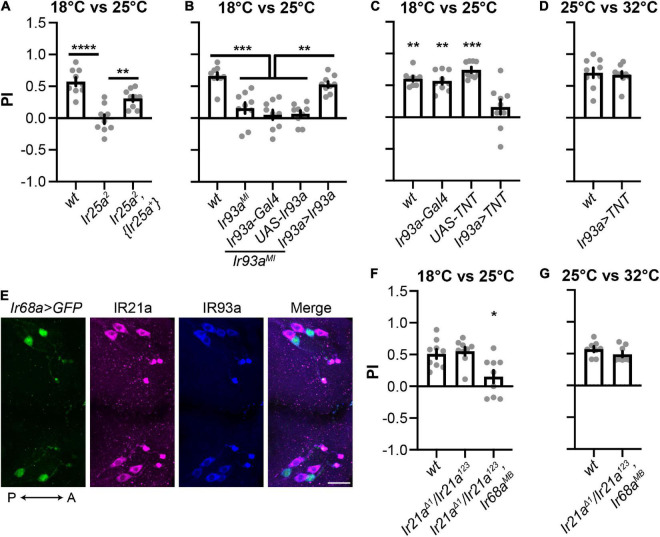
Cool and warm IR receptors are both sufficient for cool preferences between 18°C and 25°C. **(A,B)** IR25a **(A)** and IR93a **(B)** are necessary for cool preferences between 18°C and 25°C. *n* = 9; data represent means ± s.e.m; **P < 0.01, ***P < 0.001, and ****P < 0.0001, the Welch’s test of indicated groups. *wt: CS*; *Ir93a > Ir93a,Ir93a*^MI^**: *Ir93a-Gal4,Ir93a*^MI^*/UAS-Ir93a,Ir93a*^MI^**. The same set of *Ir25a*^2^ data was used as in Figure 1C. **(C,D)** *Ir93a-Gal4* positive cells are required for cool preferences between 18°C and 25°C, but not between 25°C and 32°C. *n* = 9; data represent means ± s.e.m; **P < 0.01 and ***P < 0.001, comparing to *Ir93a > TNT* (*UAS-TNT; Ir93a-Gal4*), the Welch’s test, except the Mann–Whitney test for the comparison of *UAS-TNT* and *Ir93a > TNT* in **(C)**. **(E)** IR93a is expressed in five neurons in each DOG: two express *Ir68a-Gal4*, and the other three express IR21a. Immunostaining of GFP (green), IR21a (magenta), and IR93a (blue). The orientation is shown by double headed arrows. A, anterior; P, posterior. Scale bars, 20 μm. *Ir68a > GFP*: *Ir68a-Gal4/UAS-GFP*. **(F,G)** The role of IR21a and IR68a in two-choice assays between 18°C and 25°C, as well as between 25°C and 32°C. *n* = 8–9; data represent means ± s.e.m; *P < 0.05, comparing PI with the corresponding *wt* (*CS*), the Welch’s test for **(F)** and the Mann–Whitney test for **(G)**.

IR25a and IR93a are co-receptor IRs for both cool and warm receptors (Knecht et al., 2016; Ni et al., 2016; Hernandez-Nunez et al., 2021). To confirm the function of IR25a and IR93a in 18°C preferences, we performed rescue experiments. An *Ir25a* genomic minigene reversed deficiencies observed in *Ir25a*^2^ (Figure 3A). Similarly, the *Ir93a^MI^* phenotype was restored by an *Ir93a* cDNA driven by *Ir93a-Gal4* (Figure 3B). These results suggest that IR25a and IR93a are necessary for the selection of 18°C.

IR25a and IR93a were co-expressed in five neurons in each DOG, which were labeled by *Ir93a-Gal4* (Figure 2G). To address the role of these five neurons, we blocked their function by using *Ir93a-Gal4* to express the synaptic neurotransmitter blocker, tetanus toxin light chain (TNT) (Sweeney et al., 1995). In the two-choice assay between 18°C and 25°C, these animals did not choose 18°C (Figure 3C), suggesting *Ir93a-Gal4* positive neurons are necessary to select 18°C. In contrast, blockage of these neurons did not affect 25°C preferences (Figure 3D), indicating *Ir93a-Gal4* positive neurons are dispensable for temperature preferences between 25°C and 32°C.

IR21a is the tuning IR for cool receptors, and IR68a for warm receptors (Knecht et al., 2016; Ni et al., 2016; Hernandez-Nunez et al., 2021). Immunostaining confirmed that neurons expressing IR93a were labeled by either an IR21a antibody or *Ir68a-Gal4* (Figure 3E). Since *Ir93a-Gal4* positive neurons drove animals to choose 18°C (Figure 3C), and neither *Ir21a^Δ1^* nor *Ir68a^MB^* showed defects in selecting 18°C (Figure 1C), we proposed that IR21a and IR68a are both sufficient for cool preferences in the two-choice assay between 18°C and 25°C. To test this hypothesis, we generated *Ir21a^Δ1^/Ir21a^123^; Ir68a*^MB^** that did not exhibit 18°C preferences (Figure 3F), supporting the notion that both IR21a and IR68a are sufficient for controlling the thermotaxis between 18°C and 25°C.

### Cool and warm receptors exert opposite effects in a gradient thermotaxis

**FIGURE 4:**
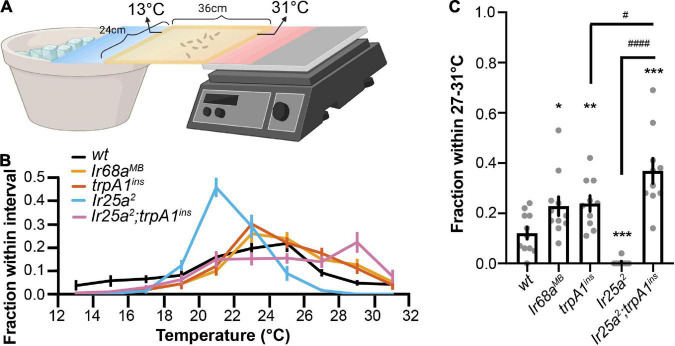
Cool and warm receptors exert opposite effects in a gradient thermotaxis. **(A)** The schematic of larval gradient thermotactic assay (created with BioRender). Early third-instar larvae were released in the middle of a testing gel at ∼22°C and were given between 10 and 15 min to make temperature selections. The number of larvae in each temperature zone was counted and the distribution was calculated as follows: (number of larvae in each temperature zone)/(total number of larvae). **(B)** Larvae distribution along a thermal gradient of indicated genotypes. **(C)** Fraction of larvae of indicated genotypes in the 27°C–31°C region. *n* = 9–11; data represent means ± s.e.m; *P < 0.05, **P < 0.01, and ***P < 0.001, comparing to *wt* (*CS*); ^#^*P* < 0.05 and ^####^*P* < 0.0001, comparing to *Ir25a^2^; trpA1*^ins^**. The Welch’s test was applied to the comparison of *wt* and *trpA1*^ins^**, *wt* and *Ir25a^2^; trpA1*^ins^**, and *trpA1*^ins^** and *Ir25a^2^; trpA1*^ins^**. The Mann–Whitney test was applied to the comparison of *wt* and *Ir68a^MB^*, *wt* and *Ir25a*^2^, and *Ir25a*^2^ and *Ir25a^2^; trpA1*^ins^**.

To address the role of cool and warm receptors in a gradient thermotaxis, we used a temperature gradient of 13°C–31°C (Tyrrell et al., 2021). Early third-instar *wt* larvae pursued 25°C in a gradient thermotactic behavioral assay (Figures 4A,B and Supplementary Video 3). TRPA1 is a component of the Rh1 pathway. The *trpA1*^ins^** mutant larvae significantly congregated in the warm region of 27–31°C, indicating a role of the Rh1 pathway in warm avoidance (Figures 4B,C). IR68a is the tuning IR receptor of warm receptors. In an optogenetic assay, IR68a, along with co-receptor IR25a and IR93a, directs avoidance behavior (Hernandez-Nunez et al., 2021). The *Ir68a^MB^* mutant larvae aggregated in a warm region of 27°C–31°C, suggesting that IR68a is necessary for warm avoidance to slow temperature changes (Figures 4B,C).

However, a similar phenomenon was not observed in *Ir25a*^2^ mutant larvae that pursued a lower temperature (Figure 4B). Since IR25a serves as a co-receptor IR for IR-formed cool and warm receptors, the *Ir25a*^2^ mutant impaired both cool and warm IR receptors but left the Rh1 pathway unaffected. As a result, the Rh1 pathway guided animals to leave warm regions and pursue a lower temperature in the *Ir25a*^2^ mutant. We tested this possibility using the *Ir25a^2^; trpA1*^ins^** double mutant that also disrupted the Rh1 pathway. *Ir25a^2^; trpA1*^ins^** mutant larvae aggregated in a warm region of 27°C–31°C (Figures 4B,C). This aggregation was significantly higher than that observed in *trpA1*^ins^** mutant larvae, supporting the function of the IR-formed warm receptor in warm avoidance to slow temperature changes.

### The role of IR92a in cool preferences between 18°C and 25°C

**FIGURE 5:**
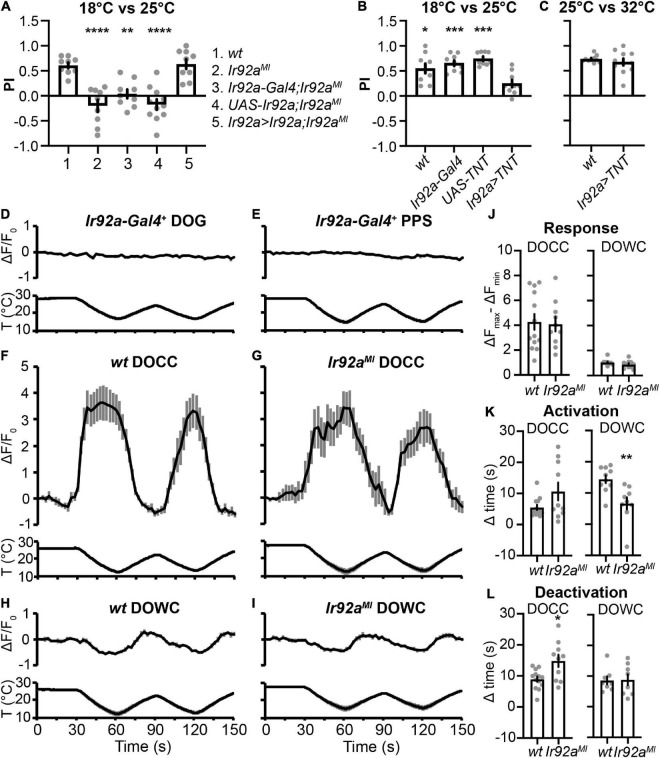
The role of IR92a in cool preferences between 18°C and 25°C. **(A)** IR92a is necessary for cool preferences between 18°C and 25°C. *n* = 9–11; data represent means ± s.e.m; **P < 0.01 and ****P < 0.0001, the Ordinary one-way ANOVA test followed by the Tukey test. *Ir92a > Ir92a,Ir92a*^MI^**: *Ir92a-Gal4,Ir92a*^MI^*/UAS-Ir92a,Ir92a*^MI^**. **(B,C)** *Ir92a-Gal4* positive cells are necessary for cool preferences between 18°C and 25°C, but not between 25°C and 32a. *n* = 9–11; data represent means ± s.e.m; *P < 0.05 and ***P < 0.001, comparing to *Ir92a > TNT* (*UAS-TNT; Ir92a-Gal4*), the Welch’s test, except the Mann–Whitney test for the comparison of *UAS-TNT* and *Ir92a > TNT* in **(B)**. The same set of *UAS-TNT* data was used as in Figure 3C. *wt*: *CS.* **(D–I)** Calcium changes in response to temperature fluctuations of indicated genotypes and cells. Fluorescence is quantified as the percent change in fluorescence intensity compared to initial intensity. *n* = 9–13; data represent means ± s.e.m. Genotypes: **(D,E)** *Ir92a-Gal4; UAS-GCaMP6m*; **(F)** *Ir21a-Gal4; UAS-GCaMP6m*; **(G)** *Ir21a-Gal4; UAS-GCaMP6m,Ir92a*^MI^**; **(H)** *Ir93a-Gal4;Ir21a-Gal80/UAS-GCaMP6m*; **(I)** *Ir93a-Gal4; Ir21a-Gal80, Ir92a*^MI^*/UAS-GCaMP6m, Ir92a*^MI^**. **(J–L)** The role of IR92a in calcium responses of DOCCs **(F,G)** and DOWCs **(H,I)**. *n* = 8–13; data represent means ± s.e.m; *P < 0.05 and **P < 0.01, the Welch’s test was applied to DOCCs’ response, DOWCs’ activation time, and DOCCs’ deactivation time; the Mann-Whitney test was applied to DOWCs’ response, DOCCs’ activation time, and DOWCs’ deactivation time.

*Ir92a^MI^* lost cool preferences in the two-choice assay between 18°C and 25°C but showed no defects between 25°C and 32°C (Figures 1C,D). Additionally, *Ir92a-Gal4* was not expressed in DOG temperature-sensing neurons (Figure 2I). To address the role of IR92a in temperature responsiveness, we first performed rescue experiments. *Ir92a^MI^* defects were reversed by using *Ir92a-Gal4* to express an *Ir92a* cDNA (Figure 5A), suggesting IR92a is necessary for the selection of the cool temperature between 18°C and 25°C. Then, we blocked the function of *Ir92a-Gal4* positive neurons by using *Ir92a-Gal4* to express TNT (Sweeney et al., 1995). These animals did not choose 18°C (Figure 5B), suggesting these neurons are necessary for cool preferences between 18°C and 25°C. In contrast, blockage of *Ir92a-Gal4* positive neurons did not impair 25°C preferences between 25°C and 32°C (Figure 5C), indicating a dispensable role of these neurons in this assay. Finally, we expressed a genetically encoded calcium indicator GCaMP6m using *Ir92a-Gal4* to address whether *Ir92a-Gal4* positive neurons respond to temperature changes (Chen et al., 2013). Neither DOG nor PPS *Ir92a-Gal4* positive neurons exhibited temperature responsiveness (Figures 5D,E).

Calcium imaging was also performed to assess the effects of IR92a on physiological responses of DOCCs and DOWCs to temperature changes. We expressed GCaMP6m in DOCCs and DOWCs using a DOCC-specific driver *Ir21a-Gal4* and a DOWC-specific driver *Ir93a-Gal4; Ir21a-Gal80*. We used the difference between the maximum and minimum fluorescence values during the first temperature cycle (30–90 s) to represent response amplitudes. To quantify DOCCs’ activation, we calculated the time it took a DOCC from the start of cooling to reach 50% of its maximum fluorescence intensity during the first cooling period (30–60 s). We quantified DOCCs’ deactivation by calculating the time it took a DOCC’s intensity to drop to 50% of its maximum fluorescence intensity from the start of warming during the first warming period (60–90 s). DOWCs’ activation during the first warming period (60–90 s) and deactivation during the subsequent cooling period (90–120 s) were quantified in the same way. *Ir92a^MI^* did not affect their response amplitudes (Figures 5F–J). However, DOWCs’ activation was significantly faster, and DOCCs’ deactivation was significantly slower in *Ir92a^MI^*, suggesting IR92a plays a role in regulating temperature responses of DOWCs and DOCCs (Figures 5F–I,K,L).

## Discussion

This study demonstrated the functional importance of IR-formed cool and warm receptors using different thermotactic assays. When a sudden temperature change occurred in the two-choice assay between 18°C and 25°C, both cool and warm IRs were sufficient for guiding animals to cool regions. These IR receptors were dispensable in temperature preferences between 25°C and 32°C. When animals were exposed to a shallow temperature gradient, cool IRs directed animals to avoid cool temperatures, while warm IRs guided them to leave warm regions (Supplementary Table 1).

The responses to slow and fast temperature increases have been studied in adult flies, which depend on distinct warm receptors and neural circuits to detect different rates of temperature change (Hamada et al., 2008; Ni et al., 2013). This study discovered that IR-formed cool and warm receptors contribute to detecting both fast and slow temperature stimuli (Supplementary Table 1). In addition, the Rh1 cascade and cool/warm IRs might function in the same pathway to control the behavioral responses to fast temperature stimuli since *Ir25a^2^; trpA1*^ins^** did not cause more severe defects in the two-choice assay between 18°C and 25°C compared to *Ir25a*^2^ and *trpA1*^ins^** (Supplementary Figure 2). However, the Rh1 cascade and warm IRs might regulate the gradient thermotaxis through distinct pathways. When both warm IR receptors and the Rh1 pathway were disrupted, *Ir25a^2^; trpA1*^ins^** mutant larvae aggregated in a warm region of 27°C–31°C significantly more than either *Ir25a*^2^ or *trpA1*^ins^** (Figures 4B,C). It is unclear how temperature information from the Rh1 pathway and cool/warm IRs is integrated in response to different rates of temperature variation.

Unlike adult flies who always select ∼25°C, fly larvae search for different temperatures when confronted with gradient and sudden temperature changes. They choose ∼18°C when exposed to a sudden temperature increase, but slowly pursue ∼25°C on a shallow temperature gradient (Hamada et al., 2008; Kwon et al., 2008, 2010; Gallio et al., 2011; Shen et al., 2011; Ni et al., 2013; Sokabe et al., 2016; Frank et al., 2017; Simões et al., 2021; Tyrrell et al., 2021; Huda et al., 2022). Further studies are needed to understand why and how larvae select a low temperature in response to fast temperature variations but a higher temperature to slow changes.

Optogenetics demonstrates DOCCs drive avoidance behavior (Klein et al., 2015; Hernandez-Nunez et al., 2021; Tyrrell et al., 2021). Cool IRs guide animals to avoid cool temperatures on a shallow gradient (Knecht et al., 2016; Ni et al., 2016; Tyrrell et al., 2021). However, they contribute to guiding animals to leave 25°C and move toward 18°C in the two-choice assay (Figure 3F), suggesting DOCCs may drive an attractive behavior in response to a sudden temperature increase from 18°C to 25°C. Moreover, both warm and cool IRs are sufficient for 18°C preferences between 18°C and 25°C. Given that these IRs respond to temperature changes and convert temperature information to electric signals, such sufficiency may indicate a potential integration, at the periphery or in the brain, of neural signals generated by warm and cool IRs. The cellular and neural mechanisms of this phenomenon need further investigation.

In adult flies, IR92a is expressed in ac1 coeloconic olfactory sensory neurons (OSNs) and functions in detecting ammonia or other amines (Benton et al., 2009). Co-receptor IRs, IR25a, and IR76b, play a redundant role in IR92a-mediated amine responses (Vulpe and Menuz, 2021). *Ir92a-Gal4* is expressed in larval DOG, DPS/DPO, and PPS (Sanchez-Alcaniz et al., 2018). In our hands, the penetrance of its expression in DPS/DPO was low—only one out of 14 DPS/DPO from seven animals contains a single *Ir92a-Gal4* positive cell. *Ir92a-Gal4* PPS neurons expressed IR25a (Figure 2J), suggesting IR25a may serve as its co-receptor IR. However, *Ir92a-Gal4* DOG neurons did not express IR25a (Figure 2I), in which IR76b may act as its co-receptor IR. Although IR92a was necessary for cool preferences between 18°C and 25°C (Figures 1C, 5A), *Ir92a-Gal4* positive neurons did not show temperature responsiveness by calcium imaging (Figures 5D,E). Unexpectedly, IR92a contributed to regulating the activation of DOWCs and the deactivation of DOCCs (Figures 5F–L) with an unknown mechanism.

In summary, we discovered that cool and warm IRs function together, but in different collaborative ways, to guide thermotactic behaviors in response to fast and slow temperature changes. We also discussed a potential role of IR92a in temperature preferences. It controls the selection of cool temperatures between 18°C and 25°C, but not between 25°C and 32°C, probably through regulating temperature responses of cool and warm neurons in DOGs. Several questions are raised for future studies, including how temperature information from multiple thermosensory pathways is integrated, why and how larvae choose different temperatures in response to fast and slow temperature variations, and whether and how other IRs regulate thermosensation. These investigations will enable us to further understand thermosensory systems at the molecular, cellular, and circuit levels.

## Data availability statement

The datasets presented in this study can be found in online repositories. The names of the repository/repositories and accession number(s) can be found at: https://doi.org/10.7910/DVN/YHV4UU.

## Author contributions

AO and LN: conceptualization, methodology, formal analysis, data curation, and visualization. AO and HB: software. AO, HB, and LN: validation and writing—original draft. AO, HB, ES, JT, JW, and LN: investigation and writing—review and editing. LN: resources, supervision, project administration, and funding acquisition. All authors contributed to the article and approved the submitted version.

## Supplementary material

Supplementary Figure 1 Switch of temperature sides does not affect cool preferences of *wild type CS* larvae between 18°C and 25°C. *n* = 8; data represent means ± s.e.m.

Supplementary Figure 2 The *Ir25a^2^; trpA1*^ins^** double mutant displays similar cool preferences between 18°C and 25°C to *Ir25a*^2^ and *trpA1*^ins^**. *n* = 9; data represent means ± s.e.m; ^**^*P* < 0.01; comparing to *Ir25a^2^; trpA1*^ins^**; the Welch’s test. *wt: CS*. The same sets of *Ir25a*^2^ and *trpA1*^ins^** data were used as in Figure 1C.

Supplementary Video 1 The temperature preference of *wild type CS* larvae in the two-choice assay between 18°C and 25°C.

Supplementary Video 2 The temperature preference of *wild type CS* larvae in the two-choice assay between 25°C and 32°C.

Supplementary Video 3 The temperature preference of *wild type CS* larvae in the gradient assay.
