# Supplementary material for: Cool and warm ionotropic receptors control multiple thermotaxes in Drosophila larvae
Source: Front Mol Neurosci. 2022 Nov 14;15:1023492. doi: 10.3389/fnmol.2022.1023492 (PMC9701816; doi:10.3389/fnmol.2022.1023492)
Supplement: Supplementary file 1 [file Data_Sheet_1.PDF]

Table S1. The Rh1 pathway and IRs expressed in DOGs regulate different thermotactic behaviors in early third-instar larvae.

		Two-choice assay		Gradient assay	Reference
		18 vs 25°C	25 vs 32°C	13-31°C	
Rh1 pathway	Rh1	×	×	#	This study and (Shen et al., 2011; Sokabe et al., 2016)
	Gq	×	×		This study and (Kwon et al., 2008; Shen et al., 2011)
	PLC	×	×		This study and (Kwon et al., 2008; Shen et al., 2011)
	TRPA1	×	×	×	This study and (Kwon et al., 2008; Kwon et al., 2010; Shen et al., 2011)
IRs	IR76b	×	×		This study
	IR25a	×		×	This study and (Tyrrell et al., 2021)
	IR93a	×		×	This study and (Tyrrell et al., 2021)
	IR92a	×			This study
	IR21a	*		×	This study and (Tyrrell et al., 2021)
	IR68a	*		×	This study

× The indicated proteins function in the behavioral assays.
# Sokabe et al. have reported that a *Rh1* mutant selects a warm region in a gradient assay between 18 and 28°C.
* The double mutant of *Ir21a* and *Ir68a* displays defects in selecting 18°C.
Orange shows indicated mutant larvae congregate in a warm region and blue in a cool region.

Reference:

Kwon, Y., Shen, W.L., Shim, H.S., and Montell, C. (2010). Fine thermotactic discrimination between the optimal and slightly cooler temperatures via a TRPV channel in chordotonal neurons. *J Neurosci* 30(31)**,** 10465-10471. doi: 10.1523/jneurosci.1631-10.2010.

Kwon, Y., Shim, H.S., Wang, X., and Montell, C. (2008). Control of thermotactic behavior via coupling of a TRP channel to a phospholipase C signaling cascade. *Nat Neurosci* 11(8)**,** 871-873. doi: 10.1038/nn.2170.

Shen, W.L., Kwon, Y., Adegbola, A.A., Luo, J., Chess, A., and Montell, C. (2011). Function of rhodopsin in temperature discrimination in Drosophila. *Science* 331(6022)**,** 1333-1336. doi: 10.1126/science.1198904.

Sokabe, T., Chen, H.C., Luo, J., and Montell, C. (2016). A Switch in Thermal Preference in Drosophila Larvae Depends on Multiple Rhodopsins. *Cell Rep* 17(2)**,** 336-344. doi: 10.1016/j.celrep.2016.09.028.

Tyrrell, J.J., Wilbourne, J.T., Omelchenko, A.A., Yoon, J., and Ni, L. (2021). Ionotropic Receptor-dependent cool cells control the transition of temperature preference in Drosophila larvae. *PLoS Genet* 17(4)**,** e1009499. doi: 10.1371/journal.pgen.1009499.
