# Supplementary figures and images for: Cool and warm ionotropic receptors control multiple thermotaxes in Drosophila larvae
Source: Front Mol Neurosci. 2022 Nov 14;15:1023492. doi: 10.3389/fnmol.2022.1023492 (PMC9701816; doi:10.3389/fnmol.2022.1023492)

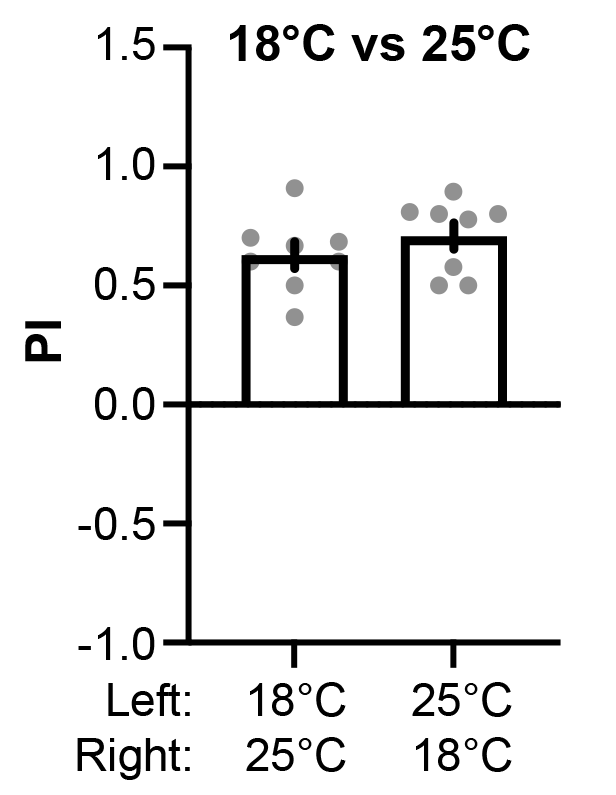

Supplement: Supplementary Figure 1 — Switch of temperature sides does not affect cool preferences of wild type CS larvae between 18°C and 25°C. n = 8; data represent means ± s.e.m. [file Image_1.TIF]

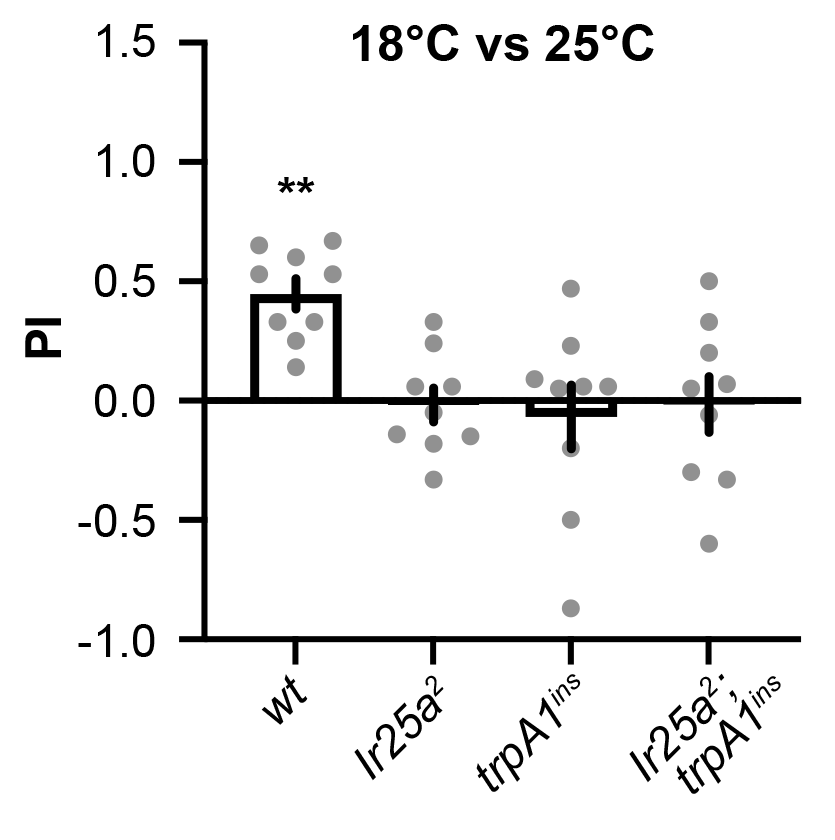

Supplement: Supplementary Figure 2 — The Ir25a2; trpA1ins double mutant displays similar cool preferences between 18°C and 25°C to Ir25a2 and trpA1ins. n = 9; data represent means ± s.e.m; **P < 0.01; comparing to Ir25a2; trpA1ins; the Welch’s test. wt: CS. The same sets of Ir25a2 and trpA1ins data were used as in Figure 1C. [file Image_2.TIF]
